# Pre- and Post-Microsurgical Rehabilitation Interventions and Outcomes on Breast Cancer–Related Lymphedema: a Systematic Review

**DOI:** 10.1007/s11912-023-01439-9

**Published:** 2023-07-04

**Authors:** David Doubblestein, Elizabeth Campione, Julie Hunley, Mark Schaverien

**Affiliations:** 1 Department of Physical Therapy, A.T. Still University, Mesa, AZ USA; 2 Physical Therapy Program, Midwestern University, Downers Grove, IL USA; 3 Department of Occupational Therapy, Mount Mary University, Milwaukee, WI USA; 4 Department of Plastic Surgery, University of Texas, MD Anderson Cancer Center, Houston, TX USA

**Keywords:** Breast cancer–related lymphedema, Microsurgery, Rehabilitation

## Abstract

**Purpose of Review:**

Breast cancer–related lymphedema (BCRL) is a debilitating progressive disease resulting in various impairments and dysfunctions. Complete decongestive therapy embodies conservative rehabilitation treatments for BCRL. Surgical procedures performed by plastic and reconstructive microsurgeons are available when conservative treatment fails. The purpose of this systematic review was to investigate which rehabilitation interventions contribute to the highest level of pre- and post-microsurgical outcomes.

**Recent Findings:**

Studies published between 2002 and 2022 were grouped for analysis. This review was registered with PROSPERO (CRD42022341650) and followed the PRISMA guidelines. Levels of evidence were based upon study design and quality. The initial literature search yielded 296 results, of which, 13 studies met all inclusion criteria. Lymphovenous bypass anastomoses (LVB/A) and vascularized lymph node transplant (VLNT) emerged as dominant surgical procedures. Peri-operative outcome measures varied greatly and were used inconsistently. There is a dearth of high quality literature leading to a gap in knowledge as to how BCRL microsurgical and conservative interventions complement each other.

**Summary:**

Peri-operative guidelines are needed to bridge the knowledge and care gap between lymphedema surgeons and therapists. A core set of outcome measures for BCRL is vital to unify terminological differences in the multidisciplinary care of BCRL.

**Condensed Abstract:**

Complete decongestive therapy embodies conservative rehabilitation treatments for breast cancer-related lymphedema (BCRL). Surgical procedures performed by microsurgeons are available when conservative treatment fails. This systematic review investigated which rehabilitation interventions contribute to the highest level of pre- and post-microsurgical outcomes. Thirteen studies met all inclusion criteria and revealed that there is a dearth of high quality literature leading to a gap in knowledge as to how BCRL microsurgical and conservative interventions complement each other. Furthermore, peri-operative outcome measures were inconsistent. Peri-operative guidelines are needed to bridge the knowledge and care gap between lymphedema surgeons and therapists.

## Introduction

Breast cancer is highly prevalent with nearly 4 million women in the USA having a history of this disease in 2019 [1]. Secondary lymphedema affects approximately 20% (range 10–50%) of patients treated for breast cancer mainly involving iatrogenic sequelae of axillary lymph nodal dissection and/or axillary lymph nodal irradiation [2–4]. Secondary lymphedema presents as an abnormal accumulation of protein-rich interstitial fluid mainly in the suprafascial tissues [5]. Breast cancer–related lymphedema (BCRL) is a debilitating progressive disease resulting in various impairments and dysfunctions including, but not limited to, swelling of the upper extremity and ipsilateral trunk due to edema, limited range of motion (ROM), decreased sensation, pain, indurated tissues, erysipelas, psychosocial distress, and decreased quality of life [6–8]. BCRL is classified by stages according the International Society of Lymphology (ISL) [9••] or by other guidelines such as the Common Terminology Criteria of Adverse Events (CTCAE) for both edema and fibrosis [10] and the Upper Extremity Lymphedema Index (UEL) (Table 1) [11]. Other diagnostic tools that assist in staging BCRL include lymphoscintigraphy, magnetic resonance lymphography (MRL), and indocyanine green (ICG) fluorescence imaging.

**Table 1:** Various models of staging lymphedema

International Society of Lymphology Staging [46]	
0	Latent or subclinical lymphedema
I	Lymphedema which subsides with limb elevation
IIa IIb	Lymphedema does not subside with limb elevation, pitting edema is present Pitting edema is difficult to present. Fibrosis and adiposity is proliferative
III	Lymphostatic elephantiasis. Pitting edema is absent. Advance stages of adiposity, fibrosis, and dermal thickening with warty overgrowths
Common Terminology of Adverse Events - Edema [10]	
1	5–10% inter-limb discrepancy in volume or circumference at point of greatest visible difference, or swelling or obscuration of anatomic architecture
2	>10–30% inter-limb discrepancy in volume or circumference at point of greatest visible difference, or swelling or obscuration of anatomic architecture, or obliteration of skin folds, or readily apparent deviation from normal anatomic contour, or limiting instrumental activities of daily living
3	>30% inter-limb discrepancy in volume or gross deviation from normal anatomic contour, or limiting self-care activities of daily living
Common Terminology of Adverse Events - Fibrosis [10]	
1	Mild induration, able to move skin parallel to plane and perpendicular to skin
2	Moderate induration, able to slide skin, unable to pinch skin, or limiting activities of daily living
3	Severe induration, unable to slide or pinch skin, or limiting joint or orifice movement, or limiting self-care
Upper Extremity Lymphedema Index [11]	
Formula	Circumference_1_^2^ + Circumference_2_^2^ + Circumference_3_^2^ + etc. Body Mass Index
Mild	Index less than 130
Moderate	Index 130 to 150
Severe	Index greater than 150

Conservative treatments for BCRL include manual lymphatic drainage (MLD), compression bandaging and garments, exercise, and skin care, which are often provided collectively as complete decongestive therapy (CDT). CDT is considered the mainstay of BCRL conservative treatment and is conducted in a decongestive phase, which is provided by a certified lymphedema practitioner, and a maintenance phase, which is conducted as self-care by the patient [12•]. Sequential pneumatic compression and elastic taping are additional modalities that compliment CDT in the management of BCRL [13]. While CDT offers benefits of reducing edema and indurated tissues, decreasing pain, improving quality of life, and slowing the progression of the disease, CDT is also limited in that it does not offer a cure for lymphedema and can be burdensome for the patient in cost, compliancy, and maintenance [14, 15•, 16].

Surgical options are available when conservative treatment fails to reduce swelling of tissues and/or improve impairment and functional goals of the patient, and can reduce the risk of future episodes of cellulitis. Physiological procedures performed by plastic and reconstructive microsurgeons include supermicrosurgical lymphovenous bypass anastomoses (LVB/A), which reduces accumulation of lymphatic fluid by anastomosis between the lymphatic vessels and venules of the venous system, and vascularized lymph node transplant (VLNT), which enables lymphangiogenesis to improve lymphatic fluid drainage in the affected extremity [17, 18, 19••]. The decision algorithm for treatment is typically based on the degree of lymphatic vessel occlusion, distribution of the lymphedema, and dermal backflow staging using ICG lymphography or lymphoscintigraphy (Table 2) [18, 19••].

**Table 2:** Cheng’s [47] grading scale for the surgical treatment of lymphedema

Grade	Symptoms	Circumferential difference	Lymphoscintigraphy	Management
0	Reversible	<9%	Partial occlusion	CDT
I	Mild	10–19%	Partial occlusion	LVB/A, SAL, CDT
II	Moderate	20–29%	Total occlusion	VLNT, LVB/A
III	Severe	30–39%	Total occlusion	VLNT + procedures
IV	Very Severe	>40%	Total occlusion	Debulking + VLNT

*CDT*, Complete decongestive therapy; *LVB/A*, Lymphovenous Bypass/Anastomoses; *SAL*, Suction Assisted Liposuction; *VLNT*, Vascularized Lymph Node Transfer

The LVB/A procedure is indicated in early lymphedema where there are linear obstructed lymphatic vessels with mild to moderate dermal backflow patterns indicating impaired transport and lymphatic fluid stasis [18, 19••]. It is a minimally invasive image-guided surgical technique using fluorescent ICG lymphography (possibly with adjunctive ultra-high frequency ultrasonographic imaging) to identify obstructed lymphatic vessels which are targeted for supermicrosurgical anastomosis to adjacent small venules. Lymphatic fluid is therefore redirected into the venous system through these bypass connections.

The VLNT procedure is indicated in advanced lymphedema where there is significant segmental dermal backflow on imaging with few or no functioning lymphatic vessels [17, 19••]. Lymph node flaps with their vascular supply are transferred from a donor site (including the axillary, inguinal, or cervical lymph node basins, or from intra-abdominal donor sites) to the axilla, forearm, or wrist of the lymphedematous upper extremity. The exact physiological response to this procedure has yet to be confirmed, but experimental and clinical studies have demonstrated both lymphangiogenesis of new afferent and efferent lymphatic collateral vessels to restore outflow as well as neo-lymphangiogenesis resulting in lymphaticovenous drainage within the transplanted lymph nodes.

Advanced chronic lymphedema is characterized by accumulation of adipose tissue, which requires direct removal by suction-assisted liposuction (SAL) or direct excisional procedures to reduce the volume [20, 21]. Studies have demonstrated that when performed axially, SAL does not impair existing lymphatic vessels and may, in fact, improve lymphatic fluid transport [20]. Despite the risks of blood loss and infections, the Charles procedure may rarely be warranted for extreme lymphedema [21].

Understanding the benefits of conservative and surgical interventions for BCRL requires that outcome measures be established. While limb volume/circumference reduction is of prime importance for microsurgeries and CDT, health-related quality of life outcomes are also an important consideration. Unfortunately, a BCRL core outcome set does not exist for clinical trials. Outcomes and instruments that measure impairments are numerous, including but not limited to circumference, volume, ROM, pain scales, strength, and sensation. Numerous patient self-report questionnaires on quality of life (QOL) exist including, but not limited to, Lymphedema Quality of Life (LYMQOL), Lymphedema International Classification of Functioning (LYMPH-ICF), and the Lymphedema Life Impact Scale [22]. The result of adjuvant CDT interventions pre- and post-microsurgery on these outcomes may vary and have not been fully investigated.

While conservative and surgical interventions for BCRL have been established and continue to be refined, these interventions seem to be at polar ends of the care spectrum for BCRL. There are limited randomized clinical trials or comparative studies on lymphatic microsurgeries that can be summarized to understand their effects. Microsurgeons often work closely with a rehabilitation team and may include certified lymphedema therapists. However, the authors hypothesized that there was a gap in the literature as to how the polar entities and interventions complement each other. The purpose of this systematic review was to investigate what rehabilitation interventions and timing of these contribute to the highest level of pre- and post-microsurgical outcomes.

## Methods

This review was registered at the International Prospective Register of Systematic Reviews on June 07, 2022 (PROSPERO, CRD42022341650) and is consistent with the guidelines of the Preferred Reporting Items for Systematic Reviews and Meta-Analysis (PRISMA) [23•].

### Literature Search

The following databases were used to search for relevant citations published from January 1, 2002 through June 1, 2022: PubMed (MEDLINE), EBSCO, and CINAHL. A combination of Medical Subject Headings (MeSH), keywords, and Boolean operators were used to search for relevant articles (Table 3).

**Table 3:** Search terms and strings

Keywords, search strings and Boolean operators	“Postoperative Care”[MeSH Terms] OR “Postoperative Complications”[MeSH Terms] OR therapy[Text Word] AND (Breast Cancer Lymphedema[MeSH Terms] OR breast neoplasms[MeSH Terms]) AND (microsurgery[MeSH Terms] OR supermicrosurgery[Text Word] OR Anastomosis, Surgical[MeSH Terms])
	“Perioperative Care”[MeSH Terms] OR preoperative[Text word] OR pre-operative[Text Word] therapy[Text Word] AND (Breast Cancer Lymphedema[MeSH Terms] OR breast neoplasms[MeSH Terms]) AND (microsurgery[MeSH Terms] OR supermicrosurgery[Text Word] OR Anastomosis, Surgical[MeSH Terms] OR Lymphaticovenous bypass OR lymphaticovenous anastomosis OR lymphovenous bypass OR lymphovenous anastomosis OR Vascularized lymph node transfer OR vascularized lymph node transplant)
MeSH = Medical Subject Headings	

### Study Selection

Two of the authors (DD and MS) independently screened the records of the comprehensive searches by titles and abstracts and then two of the authors (EC and MS) independently screened the full texts to establish the eligibility of the studies. Predetermined inclusion criteria guided the selection of studies including (1) randomized controlled trials, quasi-experimental, cohort studies, case-controlled studies, pro- and retrospective observational studies, case series, and case studies published in English with full text available; (2) human subjects with an average age 20 years or older, (3) subjects who participated in a microsurgical intervention and/or SAL for BCRL without restriction as to the description, and (4) subjects who participated in conservative interventions for BCRL pre- and/or post-operatively. Studies were excluded if lower extremity lymphedema, breast reconstruction, or gynecological cancers were the isolated topics.

### Outcomes of Interest

Primary outcomes of interest were the types of coexisting surgical and pre- and/or post-operative conservative interventions for BCRL. Secondary outcomes of interest included; (1) QoL questionnaire scores, (2) lymphedema staging, (3) circumference and/or volume measures, (4) episodes of cellulitis, and (5) adverse events.

### Quality Assessment

The methodological quality of the included studies was independently appraised by two reviewers (EC and JH) according to the Modified Downs and Black checklist [24]. The maximum Modified Downs and Black checklist score an article can receive is 28 with higher scores indicating higher quality. The two reviewers compared their independent score for each article. A third independent reviewer was available (DD) to resolve any disagreements.

### Levels of Evidence

The levels of evidence of the included studies were appraised by two reviewers (EC and JH) using the Oxford Centre for Evidence-Based Medicine: Levels of Evidence [25]. Levels range from Level 1, high-quality systematic reviews and RCTs, to Level 5, expert opinion. A third independent reviewer (DD) resolved disagreements.

### Data Extraction

Extracted data from the included studies contained the following information: author name, study characteristics (type of study, level of evidence, patient demographics), interventions (pre-operative intervention, surgical intervention, post-surgical intervention, adverse events) and post-operative outcomes (outcome measure questionnaires, lymphedema staging, circumference/volume measures, compression utilization, cellulitis infection, and MLD/CDT).

## Results

### Study Selection

**Fig. 1:**
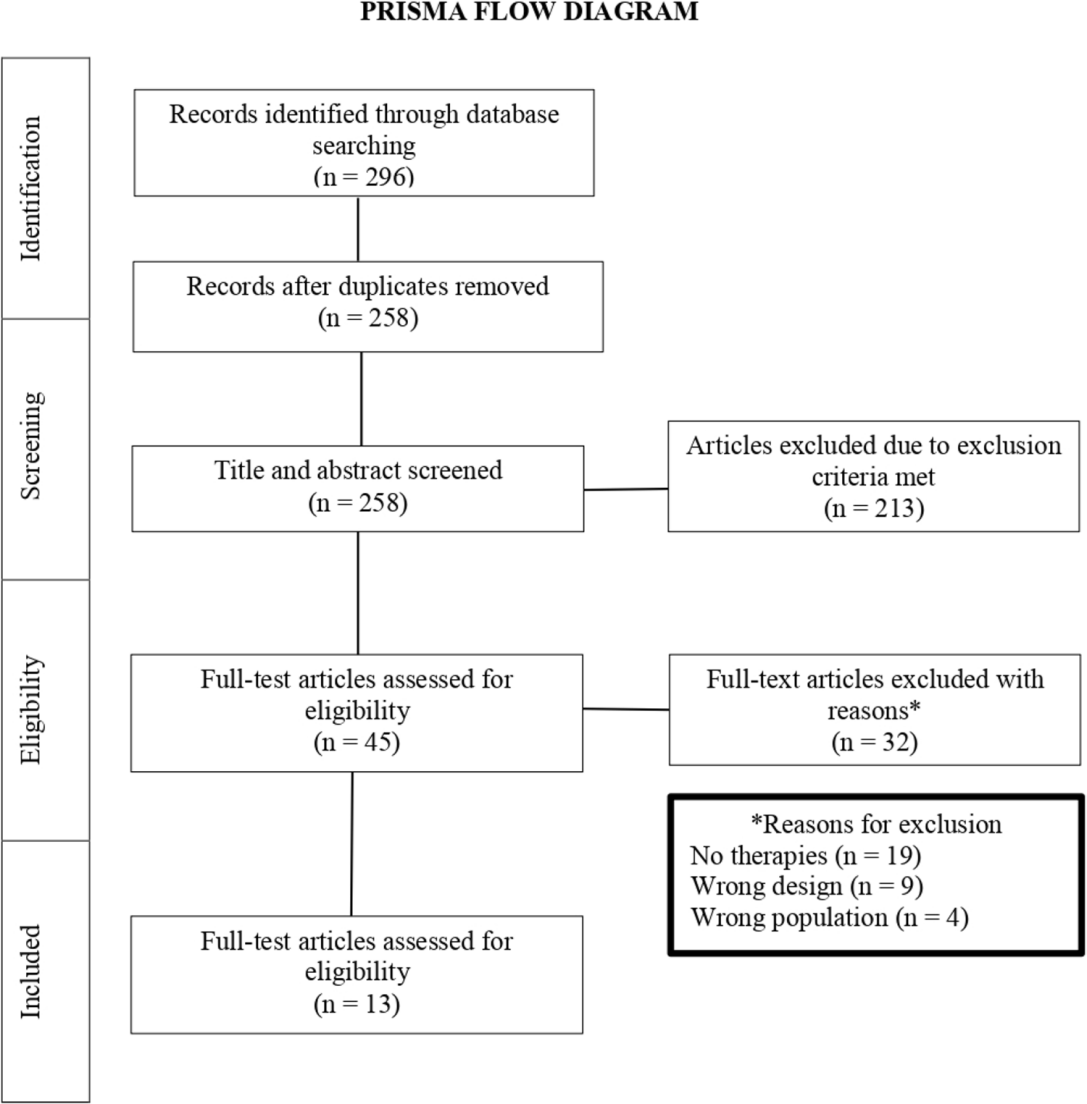
PRISMA [23•] flow chart showing screening process

The initial literature search yielded 296 results and 38 duplicates were removed. After reviewing titles and abstracts, 213 articles were removed due to not meeting inclusion criteria. Out of 45 articles that were moved forward to full text assessment, 13 studies met all inclusion criteria. The PRISMA flow chart is shown in Figure 1. Articles were excluded if they had a wrong study design, wrong population of interest, or if there were not conservative therapies mentioned in the studies. A summary of the included studies is presented in Tables 4 and 5.

**Table 4:** Study design, quality, population, methodology

Author	Design	Evidence	Quality	Patients	Pre-operative intervention	Surgery/intervention	Post-op protocol	Adverse events
Yamamoto, T., Yamamoto, N., et al.[31]	Prospective cohort	Level IV	8	8 total subjects; Lower extremity LE (*n* = 5), upper extremity LE (*n* = 3). Mean age 52.4 years. Mean BMI 23.8. Upper extremity LE mean age 45.7 years. ADB Stage of LE: III (*n* = 2), V (*n* = 1). Mean UEL for upper extremity LE before LVB/A = 141.7.		LVB/A. Mean number of LVB/As for upper extremity LE = 1.7.		
Qiu, SS, Pruimboom, T, et al.[32•]	Prospective cohort	Level IV	16	100 subjects: Male (*n* = 6), female (*n* = 94). Upper extremity lymphedema (*n* = 85), most classified as ISL stage IIA. Lower extremity lymphedema (*n* = 15). Primary LE (*n* = 6), Secondary LE (*n* = 94). Mean age of 57.1 + 10.6 years. Mean BMI = 26.3 + 4.9. Yearly cellulitis episodes for upper extremity LE (*n* = 83) = 1.0 + 1.6.	Upper Extremity LE: MLD sessions per week 1.2 + 0.8 (*n* = 70).	LVB/A with end-to-end or end-to=side anastomoses. Mean number of LVB/As per patient 2.7 + 1.4. Mean number of operations per patient 1.3 + 0.5.	No MLD or compression for 4 weeks after LVB/A. After 4 wks. subjects could restart MLD and compression as advised by specialist.	
Baumeister, RGH, Wallmichrath j, et al.[33•]	Retrospective cohort	Level IV	10	28 patients, mean age 56.3 (range 41.6–72.4). Median interval between grafting and SAL = 21 months (range 9–107).	CDT for 6 months	VLNT from the thigh donor site to upper arm or cervical recipient site. Liposuction as secondary procedure.	After grafting: flat knit compression garment used after SAL for 6 months. Garments worn 24 h/day. After SAL: elastic compression therapy continued for 6 months, then discontinued.	
Wolfs JAGN, DeJoode LGEH, et al.[26•]	Prospective cohort	Level III	15	25 females, mean age 58.4 + 8.7 years, mean BMI 25.8 + 3.7. ISL stages: I (8%), IIA (64%), III (28%). ICG stages: II (28%), III (52%), IV (20%). Mean years with lymphedema = 6.2 + 5.1 years. Episodes of cellulitis = 32. Preoperative mean volume difference using UEL was 16.2. 84% received MLD session ranging from 3×/wk. to 1× per 6 weeks with a mean of 1×/wk.		LVB/A. Mean number of anastomoses = 1.9	Did not wear compression garment or receive MLD 4 wks. PO. Afterwards with consultations MLD and/or compression could be resumed, reduced or discontinued.	
Montag E., Okada AY, et al.[27]	Prospective cohort	Level III	14	24 patients, mean age 52.6 yrs. (SD 8.89); ISL stage 1 (12.5%), 2 (62.5%), 3 (25%); Avg. duration of LE = 43.6 mos. (SD 47.62).		Breast reconstruction with VLNT (DIEP or Inguinal), 62% axilla and 37.5% wrist recipient sites		
Engel H, Lin CY, et al.[28]	Retrospective cohort	Level III	14	124 subjects divided in 2 groups 1) no breast reconstruction and 2) microvascular breast reconstruction. Mean age 55.2 + 9.4, Mean body mass 26.1 +3.9, LE 31.3 + 11.4 mos. Cheng LE grades: I (*n* = 56), II (*n* = 45), III (*n* = 20), IV (*n* = 3).		Microvascular breast reconstruction and LVB/A and VLNT	LVB/A group received 2 wks. of strengthening, 15 min of MLD 3×/day. Compression was prohibited for 1 month. Compression was resumed for 2 months and then discontinued. VLNT group received 2 weeks of strengthening, 15 min of MLD 3×/day, and no compression.	Re-exploration rate 16.9% (*n* = 21) and complication rate 8.1% (*n* = 10).
Winters H, Tielemans HJP, et al.[34]	Retrospective cohort	Level IV	15	29 females, mean age 59 + 9, median BMI = 26. Campisi stages 1b (n = X), 2a (*n* = X). Pre-OP volume difference 701 + 435 ml. 3 patients had < 10% volume difference. Avg. duration of LE = 9 + 7.3 years		LVB/A; end to end (*n* = 45), end to side (*n* = 6), invagination (*n* = 2); mean anastomoses 1.8 + 0.8).		
Gratzon A, Schultz J, et al.[29]	Prospective cohort	Level III	14	48 females, 2 males; mean age 57; ISL Stage I (16%) and II (84%); Avg. duration of LE = 4.87 years. 20% recurring cellulitis.	2-week CDT protocol	VLNT (SIEA/V, SCIA/V or contralateral thorax)	SSB daily AM and PM × 1 month. Post 1 month SSB as needed for symptoms	22% donor site, 6% recipient site, 6% both sites complications = seromas, infections, wound dehiscence, hematoma, bleeding
Dionyssiou D, Demiri E, et al.[30]	Randomized control study	Level III	14	36 subjects; all had ISL Stage II. Group A (*n* = 18) mean age 47.7 years (range 32–77), mean BMI 28.2, mean infection episodes = 1.94, mean % volume difference 36.61. Group B (*n* = 18) mean age 49.1 (range 30–71), mean BMI 27.7, mean infection episodes = 1.61, mean % volume difference 37.5.		Group A received VLNT + 6 mos PO PT. VLNT from lower abdominal and upper groin donor sites with superficial inferior epigastric or circumflex iliac artery supply. Recipient site was upper arm. Group B received PT for 6 mos.	Both groups received additional 6 mos. of PT interventions and then had no additional treatment for 12 mos. PT consisted of MLD (daily/2weeks and then 2×/wk. for 2 weeks) and compression garments (class II/ 30 mmHg) day and night. Group A also instructed to apply pumping pressure onto flap 4×10 daily for 3 mos. PO.	
Wong MM-K, Liu HL, et al.[36]	Case report	Level V	8	66-year-old female, LE duration 8 years, ISL Stage II. Bioimpedance = 54.6. Circumference = 24 cm, 20 cm, 29.5 cm, 32 cm, and 34 cm. Cumulative difference 36 cm.	6 months of lymphedema physiotherapy 3×/wk. and compression garment	VLNT with 5 nodes from groin and supplied with circumflex iliac artery and vein.	Upper limb in a semi-abducted position. Patient resumed pre-OP lymphedema physiotherapy 3x/wk. with MLD and bandaging for 4 wks. Tapered to 1/wk. for 8 more weeks. Tapered to once every 2 weeks. Discontinued physiotherapy at 4 mos. after operation.	
Chen R, Mu L, et al.[37]	Retrospective cohort	Level V	10	10 subjects, aged 36–50 years. Lymphedema duration 3 to 5 years. 4 cases of severe lymphedema and 2 cases with moderate lymphedema.		Modified microvascular lymphatic transverse rectus abdominis myocutaneous/DIEP flap accompanied with lymph nodes, lymphatic vessels, and fat.	Elastic bandages applied for 1 year.	Delayed wound healing (*n* = 1)
Chang, D, et al.[38]	Prospective cohort	Level V	7	20 females, mean age 54 years. Campisi’s LE Stage II (*n* = 10) or III (*n* = 10); mean duration of LE 4.8 years (range 1–17 years). Mean volume difference 34% (range 5–69%). 16:20 had radiation in addition to lymphadenectomy.		LVB/A bypass at distal wrist, mid-forearm, and proximal forearm performed ended to end. Mean number of bypasses 3.5 (range 2–5).	Compression bandages and elevation, and intravenous antibiotic. Continued previous compression therapy and wear compression arm sleeves 4 weeks PO.	
Yamamoto, Y, et al.[35]	Prospective cohort	Level IV	8	18 patients aged 47–80 years. 5 patients had postoperative radiotherapy. 7.1 (range 1–23) mean years since onset of edema. Circumference measured at 2 locations of the forearm. AEEC categories; Severe (*n* = 7), Moderate (*n* = 7), Mild (*n* = 4). Average enlargement difference distally 6.5 cm and proximally 7.8 cm.		LVB/A implantation combined with compression therapy. Average number of implantations 4.6 (range 3–7).	PO patients were wrapped in elastic bandage during day and elevation at night. Compression therapy continued 3 months PO. At 3 months transitioned to a 18–32 mm Hg compression sleeve garment, worn 12 h per day.	Authors report no problems with wound healing, hematoma, infection, lymphorrhea.

*LE*, Lymphedema; *BMI*, Body Mass Index; *LVB/A*, Lymphovenous Bypass/Anastomoses; *MLD*, Manual Lymph Drainage; *ICG*, Indocyanine Green; *SD*, Standard Deviation; *Avg*, Average; *AM*, morning; *PM*, evening; *MOS*, months; *PT*, Physical Therapy; mmHG, millimeters of mercury; *cm*, centimeter; *ADB*, Arm Dermal Backflow; *ISL*, International Lymphedema Society; *VLNT*, Vascularized Lymph Node Transfer; *CDT*, Complete decongestive therapy; *Pre-OP*, Pre-operatively; *PO*, Post-operatively; *DIEP*, deep inferior epigastric perforator; *SIEA/V*, Superior epigastric artery/vein; *SCIA/V*, Superior circumflex iliac artery/vein; *SAL*, Suction Assisted Liposuction; *UEL*, Upper extremity lymphedema index; *AEEC*, Average enlargement of edema circumference; *SSB*, short stretch bandage. Modified Downs and Black scale: < 7 Poor, 7–13 Limited, 14–20 Moderate, > 21 Strong

**Table 5:** Post-operative outcomes

Author	OM-Questionnaires	LE-Staging	Circ/Vol	Compression	Cellulitis	MLD/CDT
Yamamoto, T., Yamamoto, N., et al.[31]						
Qiu, SS, Pruimboom, T, et al.[32]	Mean total Lymph-ICF decrease of 13.3 points (*p* < 0.001) at mean of 25 mos. follow-up; 43.9 + 19.0 pre-OP to 30.6 + 20.2 PO. Physical function domain (*p* < 0.05) and mental function (*p* < 0.05) domain had significant score decreases.		A decrease in UEL was observed in 53% (*n* = 43) of subjects. At a mean FU of 14.4 + 3.0 mos. there was a mean difference in the UEL (pre-op:PO) of −0.4 + 8.7 (*p* = 0.787) (*n* = 40). At a mean FU of 27.5 + 4.3 mos. there was a mean difference in the UEL (pre-OP:PO) of -3.1 + 8.7 (*p* = 0.144) (*n* = 18).	Upper extremity LE (*n* = 85): 47.1% discontinued use of compression garments. 17.6% used compression garments during activities. Continuation of compression was 35.3% for upper extremity LE.	Mean decrease in yearly episodes of cellulitis (upper extremity LE *n* = 83) 0.4 + 1.0 (*p* = 0.001)	MLD sessions per week 0.8 + 0.71 (*n* = 70) (*p* < 0.001)
Baumeister, RGH, Wallmichrath j, et al.[33]			After lymphatic vessel grafting mean arm volumes decreased from 3417 + 171 cm^3^ to 3020 + 125 cm^3^ (*p*<0.001). After SAL mean arm volumes decreased from 3020 + 125 cm^3^ to 2516 + 104 cm^3^ (*p*<0.001).	37 mos. (range 7–160) after SAL 21 subjects did not require compression therapy beyond 6 months following SAL, 6 subjects used a garment continuously, 1 used garment as needed. Statistically significant difference compared to pre-OP (*p* < 0.05).		After grafting 9 subjects continued with MLD. 6 mos. after SAL, 7 subjects resumed MLD. Statistically significant difference compared to pre-op (*p* < 0.05).
Wolfs JAGN, DeJoode LGEH, et al.[26]	At 12 mos. FU, Lymph-ICF significant improvements in total score (*p* < 0.000) and in domains of hand function (*p*=0.001), mental function (*p* = 0.002), and mobility (*p* = 0.006).		Mean volume difference using UEL was 15.8 (*p* = 0.822)	At 12 mos. PO 65% of subjects completely discontinued garments, 10% wore them less often.		After 12 mos. FU 38% continued MLD at same frequencies and 38% less frequent. 24% discontinued MLD. Mean frequency of MLD was 1 time per 2 weeks.
Montag E., Okada AY, et al.[27]			Mean volume loss 20.1% (SD 44.89%) at 18 mos. PO (*p* = 0.037).		3 patients × 0.2 episodes	
Engel H, Lin CY, et al.[28]			Circumferential difference (12.8 + 4.2% vs 11.5 + 5.3%). Reduction rate (20.4 + 5.1% vs 14.7 + 6%)		Mean episodes improved from 6.2 + 1.9% to 1.9 + 1.8% (*p* = 0.03)	
Winters H, Tielemans HJP, et al.[34]	LymphQOL overall QOL improved at 6 mos. from 5.8 + 1.1 to 7.4 + 0.7 (*p* = 0.00); functionality, appearance, symptoms, and mood subdomains improved (*p* = 0.00).		Mean volume difference between arms 467 + 303 ml (*p* < 0.001) at 12 mos. PO. Volume reduction 33% at 12 mos. PO.	15 patients (53.6%) discontinued use of garments.	2 patients × 2 episodes	
Gratzon A, Schultz J, et al.[29]	LYMQOL improved at 3–12 mos. (*p* < 0.01) for function, appearance symptoms, mood, pain, heaviness, and overall QOL.		24 patients mean reduction was 58.68% (median 42.73%) at 12 mos. PO (*p* = 0.052). No intention to treat.	SSB = Avg. 76.8 h/wk. at 1-month PO, Avg. 7.3 h/wk. at 12 mos. PO. Daytime garments at 1 month PO.	2 patients × 1 episode, 1 patients × 2 episodes PO	14% discontinued all CDT therapy. 12 mos. PO daytime garments used 52.2 h/wk., alt. garments 45.2 h/wk., SSB 9.8 h/wk.
Dionyssiou D, Demiri E, et al.[30]	Visual Analog Scales at 12 mos. PO. 1. Mean pain scale decrease = Group A from 5.38 to 0.61 (*p* < 0.001), Group B from 5.22 to 4.61 (*p* = 0.077). 2. Mean heaviness decrease = Group A from 6.33 to 0.94 (*p* < 0.001), Group B 6.22 to 5.11 (*p* = 0.058). 3. Mean function improvement = Group A 5.5 to 1.22 (*p* < 0.001), Group B 5.11 to 4.61 (*p* = 0.226).		At 12 mos. PO. Mean volume Group A = 15.72 (*p* < 0.001), Group B 30.72 (*p* < 0.001). Mean volume reduction = Group A (57%) and Group B (18%)		At 12 mos. PO. Mean infection episodes = Group A 0.277 (*p* < 0.001), Group B 1.16 (*p* = 0.016)	
Wong MM-K, Liu HL, et al.[36]			Circumference 5 mos PO = 20 cm, 17 cm, 25.5 cm, 29 cm, and 32 cm. Pitting edema resolved. Bioimpedance = 22.			
Chen R, Mu L, et al.[37]			12 mos FU: upper arm circumference reductions found in 88.9% (*n* = 8). Mean reduction was 2.122 + 2.331 cm (*p* < 0.05)			
Chang, D, et al.[38]			65% of subjects (*n* = 13) had mean volume difference of 35% at 12 mos.			
Yamamoto, Y, et al.[35]	No standardized questionnaire. Patients asked about lighter limb, softer skin, fit of clothing, and frequency of lymphangitis. All patients indicated better results. 44.5% considered excellent, 33.3% considered good, 22.2 considered fair, and 0 % considered poor.		Avg. decreases in circumference were 3.7 cm (range 0–8.5 cm) at distal site and 3.6 cm (range 0.5–7.0 cm). %REC categories; Excellent (*n* = 8), Good (*n* = 6), Fair (*n* = 4), poor (*n* = 0) at an average FU period of 24 months.			

*LE*, lymphedema; *CDT*, complete decongestive therapy; *MLD*, Manual Lymph Drainage; *LYMQOL*, Lymphedema Quality of Life Questionnaire; *QOL*, Quality of Life; *Pre-OP*, preoperatively; *PO*, post-operatively; *FU*, follow-up; *mos*., months; *SAL*, Suction Assisted Liposuction; *Lymph-ICF*, Dutch Lymphedema Functioning, Disability, and Health Questionnaire; *UEL*, upper extremity lymphedema index; *%REC*, % reduction of edema circumference; *SSB*, short stretch bandage; *ml*, milliliters; *SD*, standard deviation; *cm*, centimeters

### Methodological Quality

Levels of evidence were based upon study design and quality of study [25]. The 13 included studies demonstrated variable levels of evidence with 5 at Level III [26•, 27–30], 5 at Level IV [31, 32•, 33•, 34,35], and 3 at Level V [36–38]. These results are additionally supported by the variable methodologic quality on the Modified Downs and Black checklist, ranging from 7 to 16 points out of a possible 28 (Table 4). Studies were rated by the following scores: < 7 = poor, 7–13 = limited, 14–20 = moderate, > 21 = strong [39]. Only 1 included study had poor/limited quality [38], while 5 studies were rated as limited [31, 33•, 35–37], and 7 studies were rated as moderate [26•, 27–30, 32•, 34]. The Modified Downs and Black criteria that were not met by any of the studies were items 12–15, 24–25, and 27 which encompassed representativeness of the sample and treatment, blinding of participants and study personnel, adjustment for confounding factors, and power. Few studies [29–31, 32•, 34, 36] examined participants lost to follow-up, representativeness of the entire population, and randomization into intervention groups. Many of the included studies had limitations related to external validity and selection bias.

### Population

There were a total of 453 subjects with a mean age of 55 years (range 32–80 years). Body mass index (BMI) was reported in six studies [26•, 28, 30, 31, 32•, 34] with a mean of 26. Females made up the majority of the sample population in studies that reported on sex. The duration of lymphedema prior to surgery was a mean range of 63 months (range 31.3–108 months). There was significant heterogeneity in reporting pre-operative upper extremity (UE) volumes, using UEL, volume differences, percent volume differences, bioimpedance spectroscopy, and average circumferential differences (Table 4). Lymphedema stage was identified in 12 of the studies with 6 studies [26•, 27, 29, 30, 32•, 36] reporting subjects with ISL stages I, II, III; 1 study [31] reported Arm Dermal Backflow (ADB) stages III and V, 2 studies [27, 38] reported Campisi stages 1b, 2a, 2, and 3; 1 study [28] reported Cheng stages 1–4; 1 study [35] reported average enlargement of edema circumference (AEEC) mild, moderate, and severe; and 1 study [37] reported using author-defined stages of moderate and severe (Table 4). Cellulitis prior to surgery was described by Qiu et al. [32•] and Dionyssiou et al. [30] as 1 ± 1.6 and 1.94 ± 1.6 episodes per year, respectively, whereas Wolfs et al. [26•] and Gratzon et al. [29] reported that 32% and 20% of their subjects had pre-operative cellulitis, respectively.

### Microsurgery

LVB/A [26•, 28, 31, 32•, 33•, 34, 38] and VLNT [27–30, 35–37] emerged as dominant surgical procedures being reported in the current literature. Details of the surgical interventions were not elucidated in all studies. LVB/A surgeries consisted of anastomoses that were intersusception, end-to-end, and end-to-side, and for VLNT various donor sites were utilized (axilla and inguinal) (Table 4). Pre-operative conservative interventions were reported in only 4 studies [29, 32•, 33•, 36] and varied significantly. Two studies [33•, 36] recommended 6 months of lymphedema therapy, but only Wong et al. [36] delineated specifics of 3×/week and the addition of wearing a compression garment. Another pre-operative intervention was 2 weeks of CDT [29], and yet another reported only MLD treatment at 1.2 ± 0.8 sessions per week [32•]. Post-operative protocols existed in all studies except for Yamamoto [31] Montag et al. [27] and Winters et al. [34]_._ Post-operative protocols varied widely and partially depended on the type of surgical intervention. Three studies recommended no post-operative compression for 4 weeks [26•, 28, 32•], whereas other studies used post-operative compression [29, 33•, 35–38]; however, significant variations existed with reporting of short stretch bandages, elastic bandages, garments without grades, and garments with grades of 18–32 mmHg (Table 5). Furthermore, compression usage ranged from daytime or 24-h wear and from 2 weeks to 1 year. Despite the heterogeneous use of compression, LVB/A surgeries did not use compression for 4 weeks post-operative [26•, 28, 32•], whereas VLNT used compression multifariously [29, 30, 33•, 36, 37]. MLD was recommended by a few studies [28, 30, 36] at frequencies of daily or 3×/week and then reducing frequency over time. Other studies [26•, 32•] did not use post-operative MLD interventions. Engel et al. [28] reported using strengthening interventions as part of their post-operative protocol [28]. Adverse events were reported by 4 studies [28, 29, 35, 37] (Table 4).

### Post-Operative Outcomes

There were many gaps in the literature reporting on various post-operative outcomes. Despite all studies using a method of preoperative lymphedema staging, none of the studies re-staged the subject(s) post-operatively. Most studies reported on volume and/or girth measurements; however, not all improvements were significant (Table 5). At 12 months’ post-operative follow-up, Chen et al. [37] reported a significant mean reduction in limb volume (*p* < 0.05) in 88.9% of their subjects, while Chang et al. [38] reported a mean volume difference of 35% in 65% of their subjects. Montag et al. [27] reported a significant (*p* = 0.04) mean volume reduction of 20.1% (SD 44.9%) at 18 months after VLNT. Baumeister et al. [33•] reported a significant reduction in arm volumes after lymphatic vessel grafting (reduced by 397 cm^3^, *p*<0.001) but further significant reductions were accomplished with SAL as a secondary procedure (reduced by 504 cm^3^, *p*<0.001). Although improvements in UEL occurred post-operatively, Qiu et al. [32•] and Wolfs et al. [26•] reported non-significant results; *p* = 0.14 and *p* = 0.82 respectively. Other studies reported changes in volume and/or circumference but did not delineate the significance (Table 5) [35, 36, 38]. Of interest were the mean volume reductions of the arm with post-operative physical therapy (PT) compared to those without post-operative PT in the randomized-controlled trial by Dionyssiou et al. [30]_._ The authors determined that there were greater reductions (*p*<.001) in the group receiving post-operative PT (57%) compared to without PT (18%).

Post-operative QOL was reported by six studies [26•, 29, 30, 32•, 34, 35] with LYMQOL [29, 34] and Lymph-ICF [26•, 32•] demonstrating significant improvements (*p* < 0.05) in domains of physical function and mood/mental QOL. Reduction in episodes of cellulitis was reported in 6 studies [27–30, 32•, 34], with Dionyssiou et al. [30] reporting a significant reduction for conservative treatment and surgical study groups, *p* < 0.001 and *p* = 0.02 respectively).

The abatement, reduction, or continuation of conservative lymphedema treatment was only mentioned in 5 studies [26•, 29, 32, 33•, 34]. Four studies reported on patients who discontinued compression at rates of 47.1% [32•], 75% [33•], 65% [26•], and 53.6% [34] (Table 5). In comparison, MLD continued more frequently with reports of discontinuation in only 14% [29] and 24% [26•] of the subjects.

## Discussion

This systematic review investigated rehabilitation interventions that may have led to improved levels of pre- and post-operative outcomes for patients diagnosed with BCRL. The dearth of available literature pertaining to both pre- and post-microsurgical conservative interventions confirmed our hypothesis that there was a gap in the literature as to how microsurgical and conservative interventions complement each other. Although there is clear evidence that LVB/A and VLNT with or without adjuvant SAL have demonstrated stable long-term improvements for BCRL, there is limited high quality evidence encompassing the broad spectrum of microsurgical interventions combined with pre- and post-operative conservative interventions.

This review revealed that literature for comprehensive guidelines to microsurgical peri-operative care for BCRL is scarce. In addition, despite that compression therapy appears to be the most recommended post-operative intervention [40], a guideline in its usage and dosage is lacking. Inconsistencies of post-operative rehabilitation including CDT, compression, MLD, skin care, exercises, education, and lymphedema therapist consultation were evident. Research has investigated prehabilitation (i.e. pre-operative rehabilitation) for orthopedic and cardiovascular conditions [41, 42], but has also been explored in breast cancer surgery [43••]. Studies have demonstrated that prehabilitation has been favorable in feasibility, improving post-operative functional capacity of the musculoskeletal and cardiovascular systems, fostering mental well-being, and reducing adverse post-operative outcomes [41, 43•, 44]. A study outside of our literature search date range used CDT 3 months preoperatively and 6 months post-operatively [45••]. Ciudad et al. [45••] reported that their use of preoperative CDT also helped determine the need for SAL in their algorithm. The authors concluded that CDT was essential pre- and post-operatively for improved outcomes [45••]. While the level of details for this algorithm was sufficient, it is worth observing that this study did not compare results with a control group. Notably, other peri-operative BCRL interventions not represented in the included studies may include aerobic exercise, upper quadrant resistance training, stretching and mobility exercises, dietary consultation, phytotherapeutics to soften skin, and education [42, 43•, 44]. This systematic review also revealed inconsistencies in post-operative rehabilitation, lymphedema therapist consultation, and conservative interventions. Furthermore, the reporting of outcomes was heterogeneous in type of measures, time points, and instrumentation. Currently, there is a critical need for a core set of standardized outcomes which will enable cross disciplinary reporting in clinical and research settings.

At the conclusion of this review, the authors were left with unanswerable questions. Is there a benefit for pre-operative CDT? What defines a failed CDT or conservative management of BCRL? What are the markers for pre-operative optimization (prehabilitation)? Do the outcomes of surgery reflect the pre- or post-operative conservative interventions? Are there established peri-operative guidelines to bridge the gap of knowledge and care between lymphedema surgeons and lymphedema therapists?

## Strengths and Limitations

Random error is present in this review due to the heterogeneity of the study designs and outcome measures. Many randomized controlled trials about microsurgeries for BCRL exist, but high quality literature involving conservative interventions with microsurgery as a comprehensive protocol is scarce. This lack of literature also brings the uncertainty of ascertaining all related studies in our literature search. Despite this limitation, our extensive inclusion criteria strengthened our finding that there is a gap in the literature and our twofold method for assessment of study quality strengthened our finding of modest current studies.

## Conclusions

There is a dearth of high quality literature leading to a gap in knowledge as to how BCRL microsurgical and conservative interventions complement each other. Peri-operative guidelines are needed to bridge the knowledge and care gap between lymphedema surgeons and therapists. A core set of outcome measures for BCRL is vital to unify terminological differences in the multidisciplinary care of BCRL.
